# Ranking important predictors of the need for a high-acuity psychiatry unit among 2,064 inpatients admitted to psychiatric emergency hospitals: a random forest model

**DOI:** 10.3389/fpsyt.2024.1303189

**Published:** 2024-02-08

**Authors:** Mai Iwanaga, Sosei Yamaguchi, Satoshi Hashimoto, Shimpei Hanaoka, Hiroshi Kaneyuki, Kiyoshi Fujita, Yoshiki Kishi, Toyoaki Hirata, Chiyo Fujii, Naoya Sugiyama

**Affiliations:** ^1^ Department of Community Mental Health and Law, National Institute of Mental Health, National Center of Neurology and Psychiatry, Tokyo, Japan; ^2^ Department of Psychiatry, National Hospital Organization Kumamoto Medical Center, Kumamoto, Japan; ^3^ Chiba Psychiatric Medical Center, Chiba, Japan; ^4^ Yamaguchi Prefectural Mental Health Medical Center, Yamaguchi, Japan; ^5^ Okehazama Hospital Fujita Kokoro Care Center, Toyoake-shi, Japan; ^6^ Department of Psychiatry, Okayama Psychiatric Medical Center, Okayama, Japan; ^7^ Numazu Chuo Hospital, Numazu, Japan

**Keywords:** acute, decision-making, emergency, hospitalization, inpatient, predictor

## Abstract

**Aims:**

In order to uphold and enhance the emergency psychiatric care system, a thorough comprehension of the characteristics of patients who require a high-acuity psychiatry unit is indispensable. We aimed to clarify the most important predictors of the need for a high-acuity psychiatry unit using a random forest model.

**Methods:**

This cross-sectional study encompassed patients admitted to psychiatric emergency hospitals at 161 medical institutions across Japan between December 8, 2022, and January 31, 2023. Questionnaires were completed by psychiatrists, with a maximum of 30 patients assessed per medical institution. The questionnaires included psychiatrists’ assessment of the patient’s condition (exposure variables) and the need for a high-acuity psychiatry unit (outcome variables). The exposure variables consisted of 32 binary variables, including age, diagnoses, and clinical condition (i.e., factors on the clinical profile, emergency treatment requirements, and purpose of hospitalization). The outcome variable was the need for a high-acuity psychiatry unit, scored from 0 to 10. To identify the most important predictors of the need for a high-acuity psychiatry unit, we used a random forest model. As a sensitivity analysis, multivariate linear regression analysis was performed.

**Results:**

Data on 2,164 patients from 81 medical institutions were obtained (response rate, 50.3%). After excluding participants with missing values, this analysis included 2,064 patients. Of the 32 items, the top-5 predictors of the need for a high-acuity psychiatry unit were the essentiality of inpatient treatment (otherwise, symptoms will worsen or linger), need for 24-hour professional care, symptom severity, safety ensured by specialized equipment, and medication management. These items were each significantly and positively associated with the need for a high-acuity psychiatry unit in linear regression analyses (p < 0.001 for all). Conversely, items on age and diagnosis were lower in the ranking and were not statistically significant in linear regression models.

**Conclusion:**

Items related to the patient’s clinical profile might hold greater importance in predicting the need for a high-acuity psychiatry unit than do items associated with age and diagnosis.

## 1 Introduction

Globally, psychiatric emergency care, including high-acuity units, is an important component of a comprehensive mental health care system. A high-acuity unit plays a crucial role, for example, in initial assessment and specialized care of patients experiencing acute psychiatric crises (1). Acute care is resource intensive and is financially costly (2, 3). The number of people using psychiatric emergency services has been increasing in many countries (4–6). Hence, acute psychiatric services need to be prioritized for patients who have a greater need for a high-acuity unit. However, since the problems and conditions of patients presenting with psychiatric emergencies vary widely (7), it is difficult to develop clear criteria for identifying patients who need to be transferred to the high-acuity care unit. As a result, each psychiatrist is faced with the problem of making decisions regarding hospitalization, taking into account the complex internal and external factors of each patient, with no clear criteria or process for making admission decisions (8).

Only a few studies have attempted to identify patients who require transfer to a high-acuity unit. For example, the demographic and clinical characteristics of patients admitted to acute psychiatric units were examined in quantitative studies from the United States, Germany, New Zealand, and Canada (9–12). According to these studies, hospitalization is associated with clinical severity and other characteristics, such as age and diagnosis (9–12). Regarding age, a previous study among adults have reported an association between older age and emergency psychiatric admission (9), but this was not clear for minors. The common diagnoses of acute psychiatric inpatients were schizophrenia and dementia (10, 11). Other studies have reported that patients with involuntary admission, which represents the majority of admissions to acute psychiatric units, are characterized by the presence of psychosis and a high number of various clinical problems (13). Three qualitative studies examined clinical profiles of patients admitted to acute units in more detail (8, 14, 15). For example, they included a variety of clinical profiles, such as risk of self-harm or harm to others, medical severity, and medication noncompliance (8, 15). Thus, several characteristics in previous studies have been reported as being relevant to the need for a high-acuity unit.

Despite the existing evidence, it remains unclear which of these factors are most important in determining the need for a high-acuity unit. In other words, little is known about which factors psychiatrists consider most important when deciding to admit patients to high-acuity units. In addition, the complexity and heterogeneity of patient profiles continue to impose challenges for establishing definitive criteria for admission eligibility. Two limitations of earlier studies need to be addressed. First, previous quantitative studies have focused primarily on the demographic characteristics and diagnoses of patients admitted to acute psychiatric units, with limited exploration of the detailed clinical profiles revealed in qualitative research. The factors among various elements, such as patient attributes, diagnoses, and clinical conditions, substantially influence the need for a high-acuity unit need to be identified. Second, most quantitative studies have used traditional prediction models, such as linear regression. Due to the interactions among various factors, such simple models might have limited statistical predictability and accuracy. In addition, the predictors of the need for a high-acuity unit are multiaxial, diverse, and complex. One approach to modeling the complexity of multiple clinical assessments is machine learning, such as random forest modeling (16), which is increasingly being used in mental health research (17, 18). Such models should be used to deal with complex data, such as those described above.

To fill these research gaps, we conducted an exhaustive survey of psychiatric emergency hospitals in Japan and aimed to clarify the relative importance of predictors of the need for a high-acuity psychiatric care unit by using a random forest machine learning approach. In Japan, the psychiatric emergency system was established in 1995 and is now available nationwide. In the public healthcare insurance program, a high-acuity unit is designed to discharge inpatients within 3 months. Previous studies of patients admitted to a high-acuity unit in Japan have reported that many inpatients have schizophrenia (19–22). However, no studies have analyzed the clinical profiles of inpatients in detail. Identifying the factors strongly associated with the need for a high-acuity unit could guide psychiatrists’ decision-making regarding hospitalization, which has traditionally been left to their discretion. Moreover, this process is expected to enhance the transparency and objectivity in medical care, particularly in acute psychiatric settings. Additionally, this study will contribute to policy making, including establishment of criteria to facilitate the admission of individuals with a marked need for high-acuity units, thereby sustaining a high-quality acute psychiatric care system.

## 2 Materials and methods

### 2.1 Study design and participants

We conducted a nationwide cross-sectional study of medical institutions with psychiatric emergency acute care inpatient units in Japan. We recruited all 161 medical institutions with high-acuity units across Japan, on December 8, 2022. At each medical institution, psychiatrists completed questionnaires, up to a maximum of 30 patients per institution, to prevent inclusion of a larger number of participants from a particular institution or region. The inclusion criterion was admission to a high-acuity unit from November 8, 2022, to January 31, 2023. No exclusion criteria were established. This study was approved by the Research Ethics Committee of the National Center of Neurology and Psychiatry (No. A2022-065).

### 2.2 Measures

The questionnaire used in this study was developed based on the guidelines for psychiatric emergency care from the Japanese Association for Emergency Psychiatry and discussions among experts in psychiatric emergency care (23). The questionnaires included the patient’s age, diagnosis, and psychiatrists’ assessment of the clinical condition (exposure variables) and the need for a high-acuity unit (outcome variable).

#### 2.2.1 Exposure variables

We included 32 items as exposure variables. All variables were binary (1 = applicable, 0 = not applicable).

##### 2.2.1.1 Age

To examine whether being a child or being older were predictors of the need for a high-acuity unit, age was divided into two categories: under 20 years and over 70 years. Child and adolescent psychiatry and geriatric psychiatry are considered subspecialties, with specialized services provided for each category. The age boundaries were determined based on discussions among experts, including psychiatrists.

##### 2.2.1.2 Diagnosis based on the international classification of diseases, 10th edition

The psychiatrist reported the participant’s primary diagnosis (a single diagnosis) from the following 10 items: F0–F8 and dementia, based on the ICD-10.

##### 2.2.1.3 Factors on the clinical profile

Clinical profiles included three categories (i.e., cross-sectional, longitudinal, and predictive) with eight items (**Supplementary Table 1**). Cross-sectional items were symptom severity and impact on society and family. Longitudinal items were initial onset, relapse, caregiver crisis, and lack of information. Predictive items were essentiality of inpatient treatment (otherwise symptoms will worsen or linger) and expected improvement with inpatient treatment.

##### 2.2.1.4 Factors on emergency treatment requirements

Five items on emergency treatment requirements were included (**Supplementary Table 1**). These were harm to others, self-injury, lack of autonomy, irrational refusal or lack of desire for help, and other social dysfunction.

##### 2.2.1.5 Factors on the purpose of hospitalization

Seven items on the purpose of hospitalization were included (**Supplementary Table 1**). They were 24-hour professional care, medication management, elaborate diagnosis and rapid assessment of treatment efficacy, safety ensured by specialized equipment, dedication to recuperation, specific treatment, and preservation of home function by providing respite for household members.

#### 2.2.2 Outcome variable

While the participants of this study were patients who had already been admitted to high-acuity units, the level of need for a high-acuity unit was considered to vary. In this study, the need for a high-acuity unit was considered by using a non-slider visual analog scale consisting of 0 (Not necessary) to 10 (Absolutely necessary). The analysis included people with no or a low need for a high-acuity unit, as well as people with a strong need for a high-acuity unit. Thereby, predictors of the strong need for a high-acuity unit were identified. As no objective measures of the need for a high-acuity unit or patient admission criteria exist, psychiatrists were asked to respond based on their subjective assessment. No cutoff point to separate the levels of need for a high-acuity unit exist. Therefore, in this study, the score was treated as a continuous variable.

### 2.3 Statistical analysis

To investigate the importance ranking of predictors of the need for a high-acuity unit, we used a random forest regression model (16) with hyperparameter tuning. Among the various machine learning models, the random forest model was determined to be a good fit for this study design, because it is superior in terms of determining the importance of features and reducing the risk of over-fitting, even when data uncertainty is large. For tuning, the number of variables tried at each split (mtry) with the lowest out-of-bag (OOB) error was calculated. In the random forest model, the number of variables tried at each split was set to 3 and the number of trees was set to 500. The most important variables were identified using permutation importance (increase in node purity), which was computed with OOB data to measure the prediction strength of each variable.

As a sensitivity analysis, we used multiple linear regression to investigate the associations between predictors and the need for a high-acuity unit. The variance inflation factor (VIF) was calculated to confirm multicollinearity. The Breusch–Pagan test was performed to confirm heteroscedasticity. Data with no missing values were included in all analyses. Statistical significance was set at 0.05. All analyses were conducted using R statistical software (version 4.0.3, R Foundation for Statistical Computing, Vienna, Austria). The “randomForest” package was used for analysis using the random forest model.

## 3 Results

**Figure 1:**
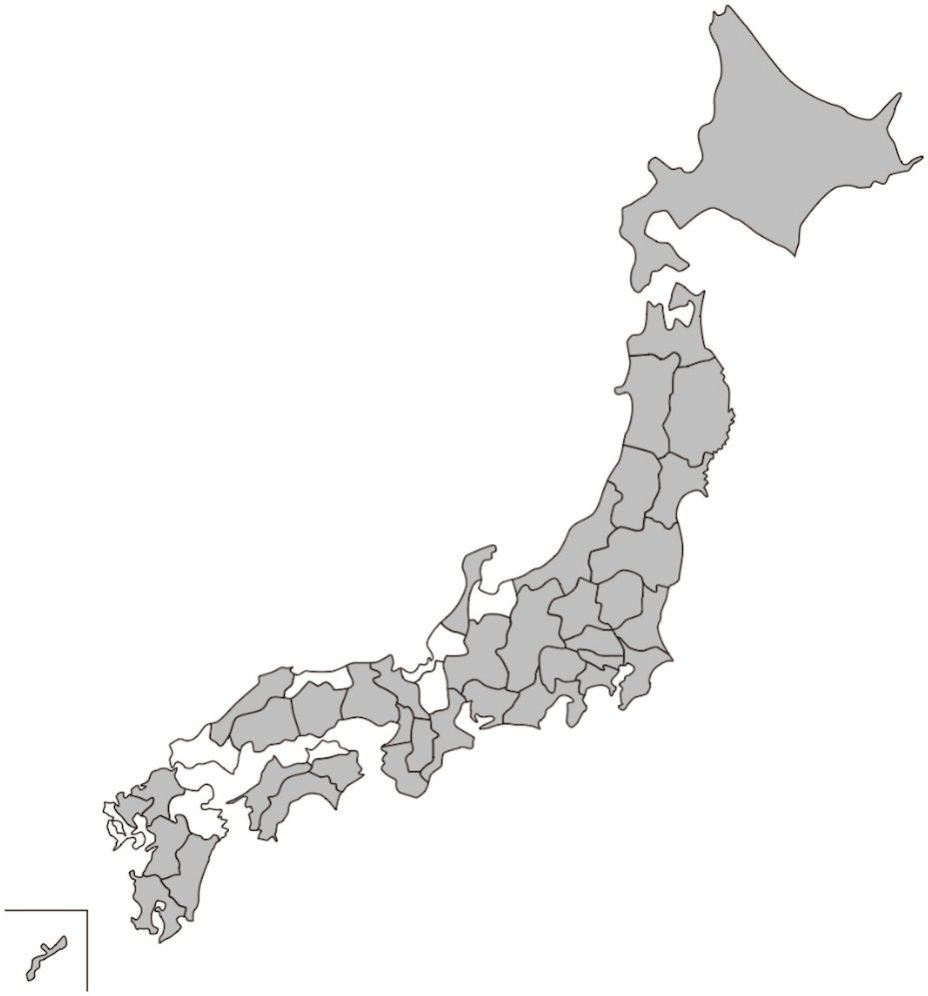
Locations of the 81 participating medical institutions.

**Figure 2:**
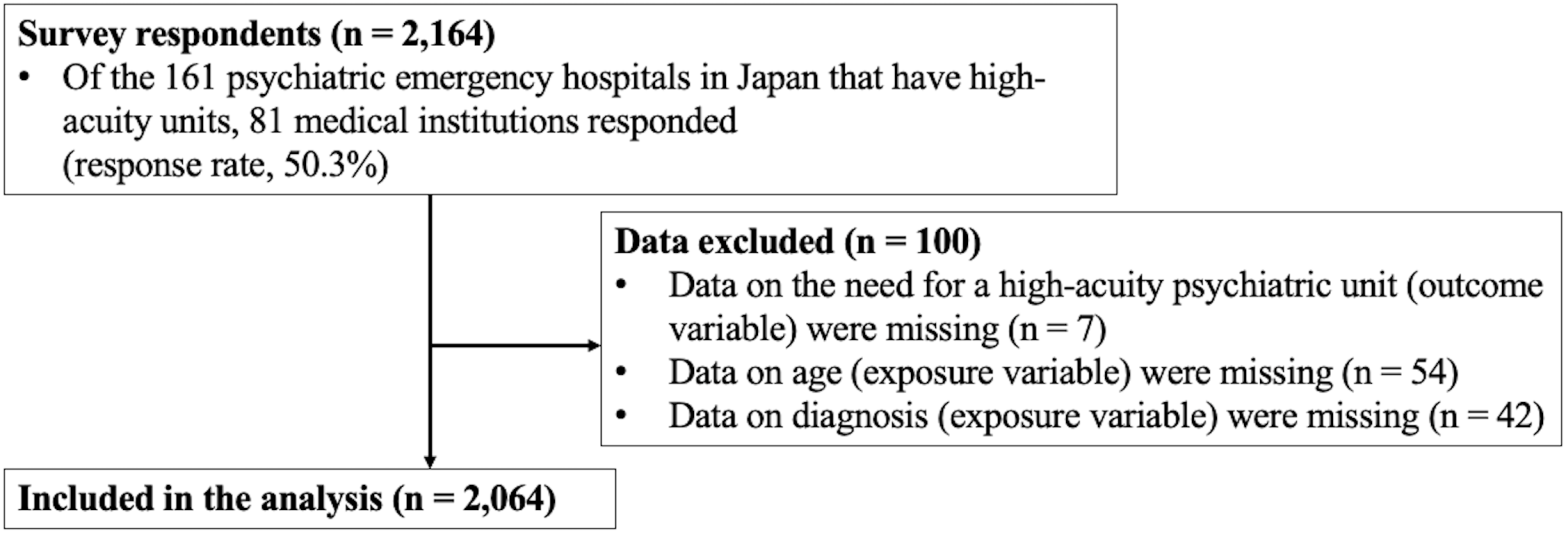
Study flow chart.

Of 161 medical institutions across Japan, 81 responded (response rate, 50.3%). Medical institutions in 40 of the 47 prefectures in Japan participated (**Figure 1**). Of the 81 participating medical institutions, 22 (27.1%) were located in designated cities, which were required to have a population of 500,000 or more. Data were collected on 2,164 patients who were admitted to high-acuity units between November 8, 2022, and January 31, 2023. After excluding patients with missing data on the need for a high-acuity unit (n = 7) or age (n = 54) and diagnosis (n = 42), 2,064 participants were included in this analysis (**Figure 2**).

**Table 1** shows descriptive statistics for the exposure and outcome variables for all participants. The most common diagnosis based on the ICD-10 was F2, schizophrenia, schizotypal, delusional, and other non-mood psychotic disorders (39.8%); followed by F3, mood or affective disorders (26.0%). The highest prevalence of exposure factors was observed in terms of the impact on society and family (71.7%), symptom severity (70.6%), and essentiality of inpatient treatment (otherwise symptoms will worsen or linger) (68.1%). For the outcome variable (i.e., the need for a high-acuity unit), the mean was 7.4 and the median was 8.0. The higher the score on the need for a high-acuity unit, the higher was the percentage of participants (0 = not necessary, 1.5%; 10 = absolutely necessary, 26.4%).

**Table 1:** Participants’ characteristics (n = 2,064).

	n	%
Age		
Under 20 years	124	6.0
Over 70 years	468	22.7
Diagnosis based on ICD-10		
F0: Mental disorders due to known physiological conditions	238	11.5
Dementia	185	9.0
F1: Mental and behavioral disorders due to psychoactive substance use	118	5.7
F2: Schizophrenia, schizotypal, delusional, and other non-mood psychotic disorders	822	39.8
F3: Mood [affective] disorders	536	26.0
F4: Anxiety, dissociative, stress-related, somatoform, and other nonpsychotic mental disorders	130	6.3
F5: Behavioral syndromes associated with physiological disturbances and physical factors	16	0.8
F6: Disorders of adult personality and behavior	21	1.0
F7: Intellectual disabilities	70	3.4
F8: Pervasive and specific developmental disorders	83	4.0
Clinical profile		
Cross-sectional: symptom severity	1457	70.6
Cross-sectional: impact on society and family	1480	71.7
Longitudinal: initial onset	434	21.0
Longitudinal: relapse	1510	43.2
Longitudinal: caregiver crisis	131	6.3
Longitudinal: lack of information	26	1.3
Predictive: essentiality of inpatient treatment (otherwise symptoms will worsen or linger)	1406	68.1
Predictive: expected to improve with inpatient treatment	1109	53.7
Emergency treatment requirements		
Harm to others	757	36.7
Self-injury	542	26.3
Lack of autonomy	938	45.4
Irrational refusal or lack of desire for help	492	23.8
Other social dysfunction	927	44.9
Purpose of hospitalization		
24-hour professional care	1396	67.6
Medication management	1378	66.8
Elaborate diagnosis and rapid assessment of treatment efficacy	589	28.5
Safety ensured by specialized equipment	699	33.9
Dedication to recuperation	793	38.4
Specific treatment	182	8.8
Preservation of home functions through respite for household members	412	20.0
Need for a high-acuity unit (outcome)		
0: Not necessary	30	1.5
1	22	1.1
2	69	3.3
3	86	4.2
4	61	3.0
5	204	9.9
6	139	6.7
7	258	12.5
8	428	20.7
9	222	10.8
10: Absolutely necessary	545	26.4

ICD-10, International Classification of Diseases 10^th^ Revision.

**Figure 3:**
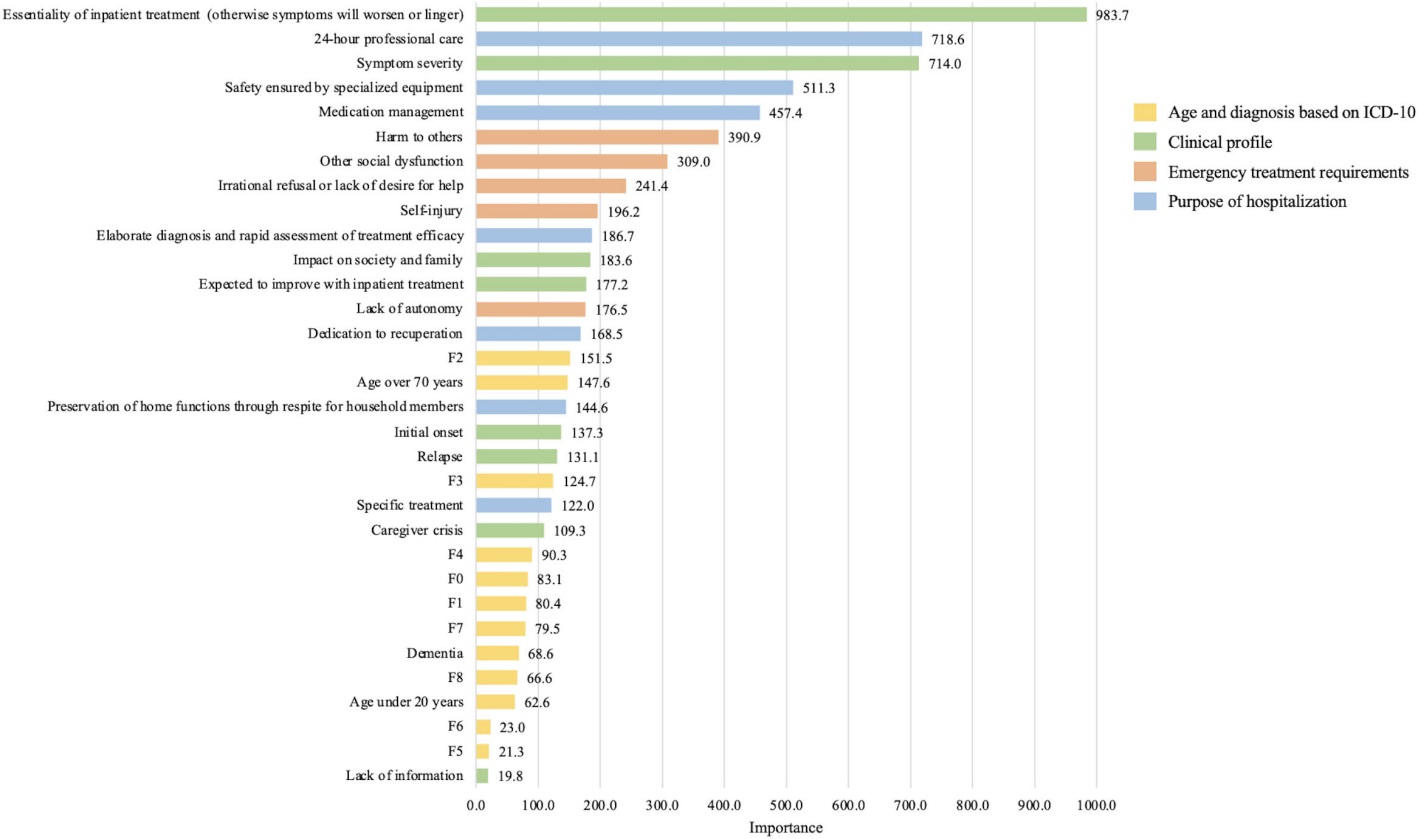
Relative importance of predictors of the need for a high-acuity psychiatric unit based on a random forest model (n = 2,064).

**Figure 3** shows the relative importance of predictors of the need for a high-acuity unit using a random forest model. In descending order of importance, the top-10 predictors were essentiality of inpatient treatment (otherwise symptoms will worsen or linger) (983.7), 24-hour professional care (718.6), symptom severity (714.0), safety ensured by specialized equipment (511.3), medication management (457.4), harm to others (390.9), other social dysfunction (309.0), irrational refusal or lack of desire for help (241.4), self-injury (196.2), and elaborate diagnosis and rapid assessment of treatment efficacy (186.7).

**Table 2** shows the associations between predictors and the need for a high-acuity unit based on a multiple linear regression model. Of the 32 exposure variables, 3 variables (i.e., F0, F2, and F3) had a VIF > 10, with heterogeneous dispersion detected by the Breusch–Pagan test. Therefore, the analysis was performed excluding F0, F2, and F3 variables. Among exposure variables, symptom severity (b = 0.63, p < 0.001), essentiality of inpatient treatment (otherwise symptoms will worsen or linger) (b = 1.10, p < 0.001), harm to others (b = 0.76, p < 0.001), self-injury (b = 0.45, p < 0.001), irrational refusal or lack of desire for help (b = 0.25, p = 0.03), other social dysfunction (b = 0.45, p < 0.001), 24-hour professional care (b = 0.88, p < 0.001), medication management (b = 0.45, p < 0.001), safety ensured by specialized equipment (b = 0.67, p < 0.001), and specific treatment (b = 0.72, p < 0.001) were positively and significantly associated with need for a high-acuity psychiatric care unit.

**Table 2:** Association between predictors and need for a high-acuity psychiatric unit based on a multiple linear regression model (n = 2,064).

	Coefficient	95% CI		p
Age				
Under 20 years	0.31	-0.08	0.70	0.12
Over 70 years	-0.22	-0.46	0.03	0.09
Diagnosis based on ICD-10				
Dementia	-0.30	-0.66	0.07	0.12
F1: Mental and behavioral disorders due to psychoactive substance use	-0.34	-0.72	0.03	0.07
F4: Anxiety, dissociative, stress-related, somatoform, and other nonpsychotic mental disorders	-0.37	-0.74	0.00	0.05
F5: Behavioral syndromes associated with physiological disturbances and physical factors	0.84	-0.16	1.83	0.10
F6: Disorders of adult personality and behavior	-0.29	-1.16	0.59	0.52
F7: Intellectual disabilities	-0.21	-0.70	0.29	0.41
F8: Pervasive and specific developmental disorders	-0.19	-0.67	0.29	0.43
Clinical profile				
Cross-sectional: symptom severity	0.63	0.39	0.87	<0.001
Cross-sectional: impact on society and family	0.12	-0.09	0.34	0.25
Longitudinal: initial onset	0.41	-0.07	0.90	0.10
Longitudinal: relapse	0.09	-0.36	0.54	0.70
Longitudinal: caregiver crisis	-0.35	-0.75	0.05	0.09
Longitudinal: lack of information	0.28	-0.60	1.16	0.54
Predictive: essentiality of inpatient treatment (otherwise symptoms will worsen or linger)	1.10	0.86	1.35	<0.001
Predictive: expected to improve with inpatient treatment	0.18	-0.02	0.38	0.08
Emergency treatment requirements				
Harm to others	0.76	0.56	0.97	<0.001
Self-injury	0.45	0.24	0.67	<0.001
Lack of autonomy	-0.02	-0.21	0.17	0.83
Irrational refusal or lack of desire for help	0.25	0.02	0.47	0.03
Other social dysfunction	0.45	0.26	0.65	<0.001
Purpose of hospitalization				
24-hour professional care	0.88	0.66	1.09	<0.001
Medication management	0.45	0.24	0.67	<0.001
Elaborate diagnosis and rapid assessment of treatment efficacy	-0.05	-0.27	0.16	0.62
Safety ensured by specialized equipment	0.67	0.47	0.87	<0.001
Dedication to recuperation	-0.07	-0.26	0.12	0.46
Specific treatment	0.72	0.41	1.03	<0.001
Preservation of home functions through respite for household members	-0.19	-0.42	0.03	0.10

CI, confidence interval; ICD-10, International Classification of Diseases 10^th^ Revision.

The analysis was performed excluding variables with a VIF of 10 or higher (i.e., F0, F2, and F3).

Adjusted R-squared value: 0.37.

## 4 Discussion

This study investigated the relative importance of predictors of the need for a high-acuity unit using a random forest model, which has not been reported previously. Our findings suggested that the most important predictor variables were not related to patient’s age or diagnosis, but rather to the patient’s clinical profile. In the linear regression model performed as a sensitivity analysis, the top-ranking items related to the patients’ clinical profile were positively and significantly associated with the need for a high-acuity unit, but age and diagnosis were not.

The characteristics of the patient’s clinical profiles, which were the top items in the ranking of predictors of the need for a high-acuity unit, are likely to have several properties. From the random forest results, the most important predictor of the need for a high-acuity unit was the essentiality of inpatient treatment (otherwise symptoms will worsen or linger). This item was associated with the need for a high-acuity unit in our linear regression model. Inherently, this item theoretically seems to apply to 100% of patients admitted to high-acuity units. Nevertheless, psychiatrists considered inpatient treatment as essential for only 68% of patients in this study. Regarding the need for a high-acuity unit, 1.5% of all participants had a score of 0 (not necessary), while 10 (absolutely necessary) was the most common score. The results indicates that not all patients admitted to high-acuity units require the specialized services offered by these wards. High-acuity units typically maintain a higher staff-to-patient ratio than do general psychiatric wards, enabling the provision of superior medical care. Consequently, in scenarios where patients or their families request higher quality medical care, among other factors, admission to these units may be considered. However, while Japan’s psychiatric reimbursement system permits admissions to high-acuity units even in cases deemed less critical, the appropriateness and efficiency of such ward operations remain debated in the field. In other words, Japanese psychiatric systems appear to influence this result, which may indicate the multifunctional and multipurpose nature of high-acuity units.

The items ranked second through fifth were intended to provide medical management for severe psychiatric symptoms via hospitalization. They included 24-hour professional care, symptom severity, safety ensured by specialized equipment, and medication management. These variables were associated with the need for a high-acuity unit in the linear regression analysis. Previous studies pointed out that severe symptoms are associated with high utilization of emergency services (9, 13, 24). To secure the lives of and benefits to such patients, intensive 24-hour care and equipment specific to psychiatric emergencies based on sufficient staff and a high standard of medical care in a high-acuity unit appear to be needed (1, 2, 15). Therefore, the top-5 factors in this study might be attributed to patients’ conditions and psychiatrists’ professional views. These findings seem to augment past evidence.

After the top-five, the next most important items were related to emergency treatment requirements related to behavioral problems and poor social functioning. The items ranked sixth through ninth were harm to others, other social dysfunction, irrational refusal or lack of desire for help, and self-injury. In particular, previous quantitative and qualitative studies have noted that psychiatric emergency inpatients are at high risk of behavioral problems, including self-harm and harm to others (7, 8, 15). In this study, these factors were observed to be important in psychiatrists’ decision-making regarding admission to high-acuity units.

The importance of items related to age and diagnosis were in the lower half of the rankings. While a prior study has reported an association between older age and high-acuity unit admissions, multiple linear regression analysis in this study, in which other clinical profile variables were entered simultaneously showed no significant association. This may be because several clinical profile variables have a greater impact on the need for a high-acuity unit than do age. The most common diagnosis in this study was schizophrenia (F2). This is consistent with data from previous quantitative and qualitative studies in Japan and abroad that have reported that most patients admitted to acute psychiatric care units have schizophrenia (11, 14, 19–22). However, an Australian study of community-based service users has shown that clinical assessments could benefit from focusing on a more complex consideration of the patient’s condition profiles, rather than on individual items, such as diagnosis (25). In particular, for psychiatric emergency service users, more complex clinical profiles, rather than simply diagnosis, might be more influential when deciding on the need for admission to a high-acuity unit, because people with schizophrenia include individuals with varying degrees of medical severity, behavioral problems, and social dysfunction. Our findings could be developed to provide a clinical and policy-making basis for further evaluation of psychiatric emergency services. For example, when evaluating the need for high-acuity units and the effectiveness of hospitalization, it may be useful to collect post-discharge data (including symptom severity and social and occupational functioning) and compare it with the pre-hospitalization characteristics identified in this study.

### 4.1 Strengths and limitations

The strength of this study was that it investigated factors important to the need for a high-acuity unit with information on patient demographics and information on the detailed clinical picture. In addition, the study was a large-scale survey of Japanese high-acuity units to identify the demographics and clinical profiles of patients newly admitted to an acute care unit. More than 2,000 responses were obtained. The sample size was sufficient for the analysis to identify predictors important to determine the need for admission to a high-acuity unit. This study would help guide psychiatrists’ decision-making regarding hospitalization and sustain a high-quality acute psychiatric care system.

This study had several limitations. First, the reliability and validity of the questionnaire items have not been verified; the questionnaire was developed based on the guidelines for psychiatric emergency care from the Japanese Association for Emergency Psychiatry (23) and discussions held by a team composed of experts in emergency psychiatric care. In particular, the outcome (i.e., need for a high-acuity unit) was based on the subjective judgment of psychiatrists, because objective criteria have not been established. Future studies might yield more convincing findings by having multiple psychiatrists review each questionnaire or by considering a few objective indicators (e.g., length of stay) in the analysis. Second, selection bias might have occurred. This survey was conducted by mail and no reminders were given. The average response rate for studies conducted in this manner is reported to be a little over 50% (26), similar to the response rate for this study. However, the analysis for this study did not include responses from approximately half of the targeted medical institutes and excluded those with missing data (n = 100). Future surveys may need to devise ways to improve the response rate. Third, although this study aimed to collect detailed clinical profile data as far as possible, other potential predictors may exist. For example, symptom severity as measured by the Positive and Negative Syndrome Scale (PANSS), a standardized instrument used in clinical settings, may be another potential predictor, particularly for patients with schizophrenia. Fourth, the results of this study do not reflect regional characteristics (population size, number of medical institutions, local resources, etc.) because the participants were drawn from medical institutions in diverse regions of Japan for the sake of generalizability. Future research should address the impact of country, region, and medical institution characteristics on factors related to the need for high-acuity units. Fifth, the multiple regression analysis that was performed as a sensitivity analysis involved heteroscedasticity, although it was necessary to examine the random forest results from multiple perspectives. Further development of statistical methods using diverse and multi-axis data, such as those used in this study, is needed in future. Sixth, the relationships between variables were unclear and clinical profile items might have duplicated content. Future studies are required to clarify the relationships among variables through network analysis or other means, with further research using only selected variables or with comparisons of various patterns.

### 4.2 Conclusion

We observed that items related to the patient’s clinical profile might be more important as predictors of the need for admission to a high-acuity unit than are items related to age and diagnosis. Further research is needed to focus on the relationships among items in a patient’s clinical profile.

## Data availability statement

Not all data are freely accessible because the participating agencies have not given informed consent for open data sharing. However, the data are available from the corresponding author upon reasonable request, following approval by the Research Ethics Committee of the National Center of Neurology and Psychiatry. Requests to access the datasets should be directed to maiiwanaga@ncnp.go.jp.

## Ethics statement

The studies involving humans were approved by The Research Ethics Committee of the National Center of Neurology and Psychiatry (No. A2022-065). The studies were conducted in accordance with the local legislation and institutional requirements. Written informed consent for participation was not required from the participants or the participants’ legal guardians/next of kin because the patients in this study are new admissions to an acute psychiatric emergency unit, the majority of whom are expected to have impaired capacity to make consent decisions, and are likely to be unable to give effective informed consent. In addition, the initial phase of acute inpatient care should focus on treatment. Since the procedure for obtaining informed consent for the study could have a negative impact on the development of the physician-patient relationship, the informed consent procedure needed to be simplified. In addition, this study is of great social significance, and the collection of data in a form that does not identify individuals is not detrimental to the participants. Therefore, the informed consent procedure was simplified, and a public document describing the study was posted in a place where participants could view it, giving them the opportunity to refuse participation if they did not wish to participate in the study.

## Author contributions

MI: Conceptualization, Data curation, Formal analysis, Investigation, Methodology, Writing – original draft. SY: Conceptualization, Investigation, Methodology, Supervision, Writing – review & editing. SaH: Conceptualization, Methodology, Supervision, Writing – review & editing. ShH: Conceptualization, Methodology, Supervision, Writing – review & editing. HK: Conceptualization, Methodology, Supervision, Writing – review & editing. KF: Conceptualization, Methodology, Supervision, Writing – review & editing. YK: Conceptualization, Methodology, Supervision, Writing – review & editing. TH: Conceptualization, Methodology, Supervision, Writing – review & editing. CF: Conceptualization, Funding acquisition, Investigation, Methodology, Project administration, Supervision, Writing – review & editing. NS: Conceptualization, Investigation, Methodology, Project administration, Supervision, Writing – review & editing.

## Supplementary material

Supplementary Table 1 Supplementary information of the factors on clinical profile, emergency treatment requirements, and purpose of hospitalization, provided in the questionnaire. mECT, modified electroconvulsive therapy.
