# Supplementary material for: Ranking important predictors of the need for a high-acuity psychiatry unit among 2,064 inpatients admitted to psychiatric emergency hospitals: a random forest model
Source: Front Psychiatry. 2024 Feb 8;15:1303189. doi: 10.3389/fpsyt.2024.1303189 (PMC10882085; doi:10.3389/fpsyt.2024.1303189)
Supplement: Supplementary Table 1 — Supplementary information of the factors on clinical profile, emergency treatment requirements, and purpose of hospitalization, provided in the questionnaire. mECT, modified electroconvulsive therapy. [file Table_1.docx]

Supplementary Table 1. Supplementary information of the factors on clinical profile, emergency treatment requirements, and purpose of hospitalization, provided in the questionnaire.

|  | **Supplementary information** |
| --- | --- |
| **Clinical profile** |  |
| Cross-sectional: symptom severity | Unable to speak and act realistically (adaptively) and rationally due to mental illness |
| Cross-sectional: impact on society and family | Serious impact on self and related others in social and family life |
| Longitudinal: initial onset | First time event |
| Longitudinal: relapse | History of previous treatment, which suddenly or gradually worsened |
| Longitudinal: caregiver crisis | Caregiver is no longer able to provide the normal support and care necessary for life due to exigent circumstances |
| Longitudinal: lack of information | Details are not known |
| Predictive: essentiality of inpatient treatment (otherwise symptoms will worsen or linger) | If inpatient treatment is not initiated, the condition will either worsen or linger |
| Predictive: expected to improve with inpatient treatment | If hospitalized, improvement can be expected. |
| **Emergency treatment requirements** |  |
| Harm to others | Fact or threat of violence, damage to property, aggressive or intimidating language or behavior, or disruptive behavior |
| Self-injury | Suicide attempts, self-injurious behavior, or strong suicidal thoughts |
| Lack of autonomy | Difficulty in feeding, hygiene, and avoiding danger due to impairment or breakdown of independence and self-defense functions |
| Irrational refusal or lack of desire for help | Difficulty in avoiding life risks due to irrational refusal, rejection, or lack of support-seeking behavior |
| Other social dysfunction | Inability to manage coherently and serious side effects due to manic, mixed states, confusion, stupor, breakdown, delirium, etc. |
| **Purpose of hospitalization** |  |
| 24-hour professional care | - |
| Medication management | - |
| Elaborate diagnosis and rapid assessment of treatment efficacy | - |
| Safety ensured by specialized equipment | - |
| Dedication to recuperation | Leaving work, housework, or other roles, etc. |
| Specific treatment | mECT, clozapine, psychoeducation, treatment programs, etc. |
| Preservation of home functions through respite for household members | - |

mECT, modified electroconvulsive therapy
